# Anesthetics may modulate cancer surgical outcome: a possible role of miRNAs regulation

**DOI:** 10.1186/s12871-021-01294-w

**Published:** 2021-03-09

**Authors:** Masashi Ishikawa, Masae Iwasaki, Atsuhiro Sakamoto, Daqing Ma

**Affiliations:** 1 Department of Anesthesiology and Pain Medicine, Graduate School of Medicine, Nippon Medical School, 1-1-5, Sendagi, Bunkyo, Tokyo, 113-8603 Japan; 2 Division of Anaesthetics, Pain Medicine and Intensive Care, Department of Surgery and Cancer, Faculty of Medicine, Imperial College London, 369 Fulham Rd, London, SW10 9NH UK

**Keywords:** MicroRNA, Anesthetics, Cancer, Anti-cancer immunity, Cell-to-cell communication

## Abstract

**Background:**

microRNAs (miRNAs) are single-stranded and noncoding RNA molecules that control post-transcriptional gene regulation. miRNAs can be tumor suppressors or oncogenes through various mechanism including cancer cell biology, cell-to-cell communication, and anti-cancer immunity.

**Main Body:**

Anesthetics can affect cell biology through miRNA-mediated regulation of messenger RNA (mRNA). Indeed, sevoflurane was reported to upregulate miR-203 and suppresses breast cancer cell proliferation. Propofol reduces matrix metalloproteinase expression through its impact on miRNAs, leading to anti-cancer microenvironmental changes. Propofol also modifies miRNA expression profile in circulating extracellular vesicles with their subsequent anti-cancer effects via modulating cell-to-cell communication.

**Conclusion:**

Inhalational and intravenous anesthetics can alter cancer cell biology through various cellular signaling pathways induced by miRNAs’ modification. However, this area of research is insufficient and further study is needed to figure out optimal anesthesia regimens for cancer patients.

## Background

Surgery is the frontline treatment of solid cancers worldwide. Over 60% of cancer patients require general anesthesia for primary surgical resection [1]. Unfortunately, most patients still die due to cancer recurrence following surgery [2]. Postsurgical death is the third most common type of death behind the death from cardiovascular disease and stroke and contributes to 7.7% of deaths globally [3] and most those patients are cancer suffers per se. Postoperative cancer recurrence often occurs in high malignancy of cancer cell phenotype but perioperative risk factors may also contribute to its recurrence. For example, surgical stress activates neural and inflammatory cellular signaling that can suppress anti-tumor immunity, increase cancer cell growth and their shedding into blood circulation, and promote cancer cell adhesion residence in remote organs, all of which contribute to tumor recurrence [4–6]. Anesthetics may be also a risk factor due to their direct immunomodulation or indirect cellular signaling effects [7]. Indeed, pre-clinical and retrospective studies indicated that some anesthetics such as inhalational agents may promote cancer cell growth, whereas others such as propofol and midazolam inhibit cancer cell growth and hence may be beneficial for cancer patients [8–10]. The molecular mechanisms behind these clinical findings are largely unknown.

microRNAs (miRNAs) are single-stranded and noncoding RNA molecule with 20–25 nucleotides and participate post-transcriptional gene regulation of mRNA via mRNA degradation and translational repression. In human, there are more than 1500 miRNAs but their roles in normal and pathological cellular function remain yet to know. Previous study demonstrated that miRNAs modulate various cell biology, including cell differentiation, proliferation, apoptosis, embryonic development, stress response, stem cell renewal, and metabolism [11–15].

It has been suggested that anesthetics, can both positively and negatively influence on cancer surgical outcome through miRNA changes. Previous in vitro studies showed that inhalational and intravenous anesthetics have both pro- and anti-cancer effects through various pathways of cancer cell biology, anti-cancer immunity, and cell-to-cell communication via miRNA expression changes. In this review, the effects of anesthetics effects on cancer cell phenotyping changes via miRNA modulation will be narratively summarised; their other effects on cancer cell biology through other cellular signaling pathways have been well documented recently [7, 16, 17] will be repeated again here.

## Main Body

### miRNA in oncogenesis

**Fig. 1:**
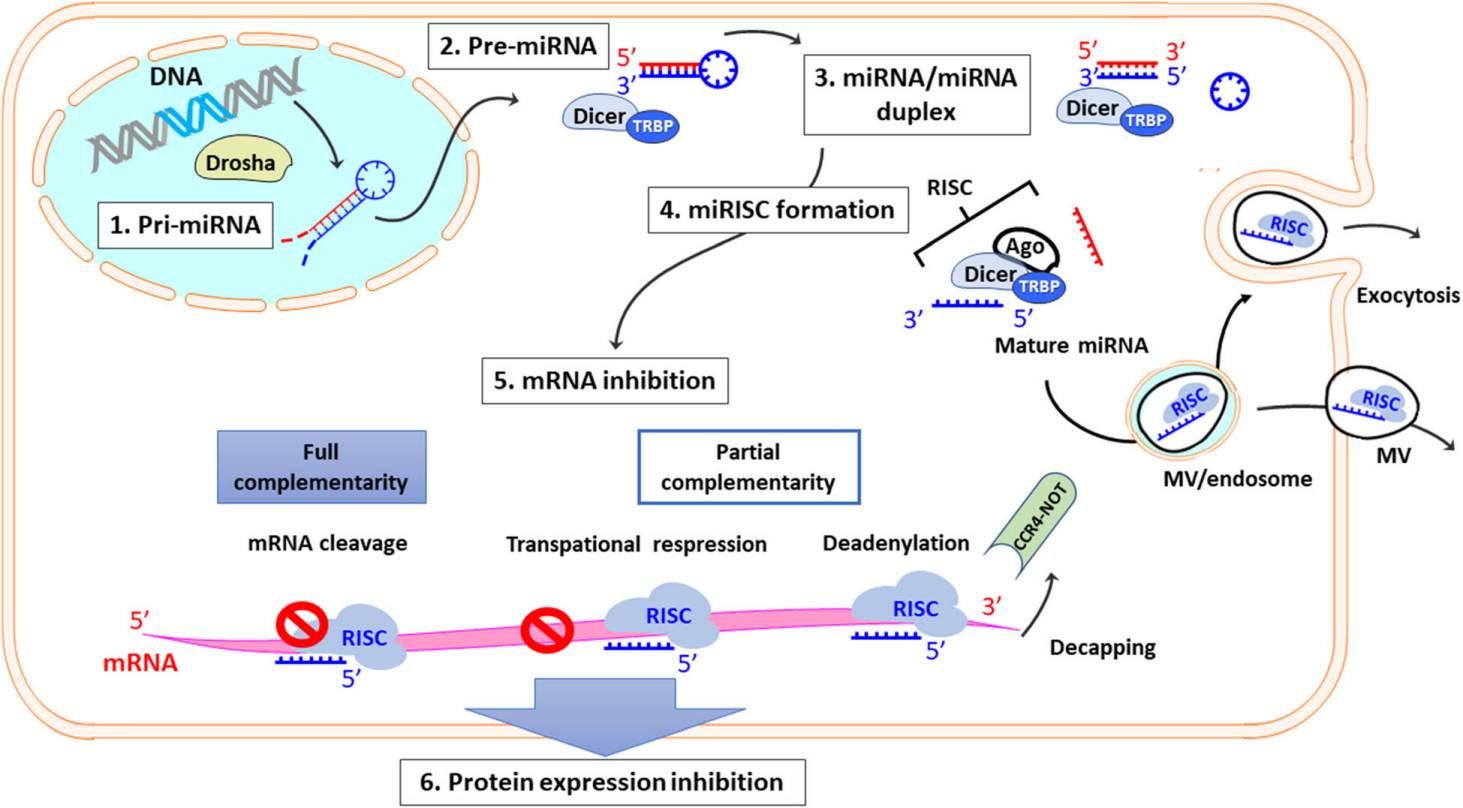
Overview of mRNA regulation by miRNA. When some stimuli including anesthesia come to the cell nucleus, pre-miRNA is made from nucleus DNA by Drosha cleavage. Pre-miRNA is cleaved by Dicer/TRBP complex and becomes mature miRNA after miRNA/miRNA duplex. miRISC is formed by Ago1–4 binding to mature miRNA, out of which Ago2 is the essential for the target mRNA cleavage. Some miRISC can move out of the cells in MV or exocytosis in the endosome. miRISC inhibits its target mRNA expressions in three ways, depending on the sequence complementarity to the target mRNA sequence; mRNA cleavage with the subsequent RNA degradation occur in full complementarity, transcriptional repression or deadenylation in partial complementarity. The target protein expressions decrease due to mRNA inhibition by miRISC, leading to cell activity suppression. miRNA: micro RNA, Pri-miRNA: primary miRNA, Pre-miRNA: precursor miRNA, TRBP: transactivation response element RNA-binding protein, RISC: RNA-induced silencing complex, miRISC: miRNA-induced silencing complex, MV: microvesicle, mRNA: messenger RNA, CCR4-NOT: carbon catabolite repression-negative on TATA-less

One miRNA can have an average of more than 100 targets [18], and multiple miRNAs can affect the expression of a single transcript target [19]. The overview of mRNA regulation of miRNA is shown in Fig. 1. Thus, minor variations in miRNA expression may have crucial consequences for malignant transformation and cancer cell activity whilst miRNA alterations may involve in the initiation and progression of human cancer [20]. miRNAs also influence on numerous oncogenesis processes, such as cellular metabolism, differentiation, proliferation, cell cycle control, apoptosis and migration [21–23]. The role of miRNAs in chronic lymphocytic leukaemia patients was reported in 2002 [24], disclosing that miRNAs are associated with the occurrence and progression of various cancers. It is known that miR-133 regulates cancer cell apoptosis with suppression of caspase-9 [25] whereas miR-24 enables cancer cells to survive by targeting X-linked inhibitor of apoptosis (XIAP) [26] which suppresses apoptosis by downregulation of caspases. miR-372 was reported to exert tumor-promoting roles and its upregulation was correlated with the tumor node metastasis stage in patients with hepatocellular carcinoma [27].

Previous studies indicated that these miRNAs could modulate cancer microenvironment or tumor transformation. miR-21 overexpression promoted tumorigenesis in prostate [28] and cervical [29] cancers. Also, miR-21 upregulation increased chemoresistance in lung adenocarcinoma [30], invasiveness and angiogenesis in renal carcinoma [31] and lymphoma [32]. miR-125a also modulated the chemo-sensitivity in breast cancer [33, 34], and promoted tumorigenesis of colon cancer [35]. miR-9 increased tumorigenesis, angiogenesis and metastasis in breast [36, 37], liver [38] and pancreas cancer [39] and squamous cell carcinoma [40]; in contrast, miR-9 was also reported to suppress angiogenesis and metastasis of melanoma [41], gastric [42] and nasopharyngeal cancers [43]. miR-455-3p enhanced invasiveness of breast cancer [44, 45], regulated the cell proliferation and migration of lung cancer [46], supressed tumorigenesis in prostate [47] and colon cancer [48, 49]. Clearly, different miRNAs have different roles in different cancers in terms of development and progression.

Early diagnosis of cancer is often difficult because of the poor sensitivity of current tumor markers but miRNAs may be expected to become early detection markers for tumors. Generally, when the pro-cancer miRNAs are highly expressed, the suppressor miRNAs show low expression. These dysregulated-miRNA expressions appear to be specific pattern of each cancer type. Therefore, the cancer-specific miRNA profiles are increasingly used in the clinical cancer diagnosis. Some circulating miRNAs are already evidenced as potential early diagnostic biomarkers in the cancers of breast [50], colorectum [51], pancreas [52], and liver [53].

### miRNA and cancer outcome

Several miRNAs are reported to be correlated to clinical cancer outcomes. However, the miRNAs can be as onco-miR or anti-onco-miR depending on cancer types. Among onco-miRs, miR-21 is widely recognized as the onco-miR in any cancer types per se. Several clinical reports revealed that the upregulation of miR-21 expression in cancer tissue and blood was positively related with chemoresistance, poor progression-free survival, worse overall survival in breast [54], pancreas [55], rectal [56], squamous cancer [57], colorectal [58], lung [59], renal cancers [60] and lymphoma [61]. miR-125 was also reported as an onco-miR among the cancer patients with squamous cancer carcinoma [62], gastrointestinal stromal tumor [63], oesophagus cancers [64], whereas it was recognised as an anti-onco-miR among cervical [65], gallbladder [66] and colorectal cancers [67]. Similarly, miR-9 was documented as an onco-miR in breast cancer [68], glioma [69] and lymphoma [70], but as anti-onco-miR in oral squamous carcinoma [71]. Also, miR-455-3p was shown to be as an onco-miR in glioma [72], but as anti-onco-miR in liver [73], breast [44] and lung cancer [46] and osteosarcoma [74]. Thus, each cancer type has its own onco-miRs and anti-miRs and each individual miRNA can be pro- and anti-cancer modulator in different cancers.

### miRNA as a therapeutic target

miRNAs also can be the therapeutic targets of cancers. One of the pivotal advantages of miRNAs as therapeutic targets is the capability of multiple regulations in several pathways, which is favourable to the efficient regulation of cancer cell biology. For example, silencing oncogenic miR-21 with antisense oligonucleotides promotes cancer cell apoptosis and suppressed proliferation in vitro, and reduced tumor mass volume in vivo [75]. Moreover, a chemotherapy agent based on a miR-34a mimic (MRX34) has reached out to the phase I clinical trial [76]. miR-34a is known as one of tumor miRNA suppressors, downregulating over 30 major oncogenes including TP-53 [77] and Programmed death-ligand 1 (PD-L1) [78, 79]. Clinical histology analysis showed that the reduced miR-34a expression in the primary tumor tissue was related with higher TP-53 expression in glioma [80], chemoresistance in breast cancer [81], and worse mortality in colon [82], prostate [83] and ovarian cancer patients [84]. From numerous in vitro studies, miR-34a can regulate vital oncogenesis processes, e.g. cancer apoptosis, chemoresistance, proliferation, migration and invasion in the cell lines of brain [77, 78, 85], esophagus [86], stomach [87], lung [88], breast [89, 90], prostate [91, 92], ovary [93] and leukaemia [94, 95]. Several in vivo studies suggested that miR-34a derivative treatment supressed tumor growth [96, 97], metastasis [98] and improved survival [99, 100]. The results of a phase I clinical trial of MRX34, miR-34a mimic confirmed the acceptable safety for 85 patients with hepatic primary/metastatic solid tumors and mild hepatic dysfunction [76]. Those patients received with several doses of MRX34 treatments (50 (*n* = 4), 70 (*n* = 16), 93 (*n* = 16) and finally 110 mg·m^− 2^ (*n* = 9)) with dexamethasone in 3 + 3 dose-escalation cohorts. The severe side effects were fever (grade 3, 4%), chills (14%), fatigue (9%), and back pain (5%). Four participants resulted in death due to bloody diarrhea with worsening respiratory dysfunction, multiple organ failure by disease progression, substantial brain metastasis, and cytokine release syndrome after bronchial hemorrhage, respectively. Biopsy evidenced the direct delivery of miR-34a to tumor cell cytoplasm by MRX34 treatment, whereas miR-34a target oncogenes were significantly suppressed in peripheral leukocytes in MRX34 dose-dependent manner (compared to pre-dose level, the combined mRNA expressions of B-cell lymphoma 2 (BCL2), DnaJ homolog subfamily B member 1 (DNAJB1), Catenin Beta 1 (CTNNB1), Forkhead box protein P1 (FOXP1) and Histone deacetylase 1 (HDAC1), MRX34 dose 50 (*n* = 4), *p* = 0.0005, 70 (*n* = 16), *p* = 0.0311, 93 (n = 16), *p* = 0.0299, 110 mg·m^− 2^ (*n* = 9), not significant)). As for the clinical disease status, MRX treatment stabilised the disease in 16 patients (24%) for 19 weeks as median (range, 11–55 weeks), but partial response confirmed in three patients (4%) and progressive disease in 31 patients (47%). Further study in terms of efficient dose and regimen is needed.

### miRNAs and their changes during perioperative period

It is widely known that many perioperative medications affected miRNA expressions [101] including and anti-coagulants. Celecoxib, one of commonly used NSAIDs (non-steroidal anti-inflammatory drugs), has been reported to inhibit cancer cell proliferation, migration, and invasion in osteosarcoma cells via miR-34a [102], and the expressions of miR-126-5p, −320a and -146a-5p were correlated with the sensitivity to celecoxib [103]. Aspirin regulated miR-155/eNOS (endothelial nitric oxide synthase) pathway and suppressed endothelial cell dysfunction under the inflammation [104]. Also, aspirin suppressed the expression of miR-24, − 191 and − 197 in plasma [105].

Surgical stress, inflammation in mucosa, epithelial and immune alteration all can be modulated by miRNA changes after anaesthesia. miR-223 was considered as a key miRNA among the anti-inflammation mechanism, which regulated the intestine macrophage differentiation and function [106]. The miR-223 upregulation was reported in the condition of the intestinal inflammation [107]. In addition, the upregulation of miR-223 was documented in acute respiratory distress syndrome/acute lung injury (ARDS/ALI) patients [108]. The miR-223 shuttling by pulmonary neutrophils to alveolar epithelial cells may be a novel therapy against ARDS [109]. Thus, if cancer patient develops ARDS after surgery, mRNA, inflammation, and immune cell changes all interacted together make patient’s conditions more complex.

Some miRNA changes can modulate the patient’s immune cell phenotype/balance changes. It has been reported that regulatory T cell immune activities were regulated by miR-125a [110], and the inflammatory T cell immunity were controlled by miR-146a expression [111]. In the lung cancer, miR-301a dysfunction led CD8+ T cell infiltration into the tumour microenvironment with the anti-tumor immune activation [112]. Also miR-582 regulated CD1B expression and dendritic cell function in the advanced lung adenocarcinoma [113], and miR-341 was reported to be related to leukocyte function [114] and immune escape [115].

Furthermore, some miRNAs have been found as potential biomarkers for perioperative organ injury including postoperative cognitive dysfunction (POCD), acute cardiac ischemia, deep venous thrombosis (DVT) and acute kidney injury (AKI) [116]. The mice model of POCD showed that miR-146a [117] or -181b-5p [118] inhibited the hippocampi inflammation and POCD development. The increased miR-122 expression in serum was found in the ischemic postconditioning [119], whereas the upregulation of miR339-5p and − 483-3p, and the downregulation of miR-139-5p in blood were documented in acute cardiac ischemia [120]. Also, the miR-1, −133a and − 499 expressions were correlated positively with pro-BNP (brain natriuretic peptide) and negatively with left ventricular ejection fraction [121]. miR-100 expression in plasma was related with the coronary plaque vulnerability [122]. It showed that the upregulation of miR-495 in plasma was related to a lower DVT possibility in a rat model [123]. In addition, miR-21 may be a biomarker of severe AKI after cardiac surgery [124, 125]. In an in vitro model, miR-146 augmented AKI via interleukin-8/CXCL (chemokine (C-X-C motif) ligand) signaling in the tubular cells [126].

### Anesthetics and miRNAs

It is known that anesthetic itself can change gene expressions. Microarray analysis in various organs showed that inhalational anesthetics affect 1.5% gene expression of 10,000 genes [127]. Sevoflurane was reported to change the expression of the circadian genes [128] and the genes encoding drug metabolizing enzymes [129]. However, molecular biological research utilizing proteomics did not identify an association of anesthetics induced gene and protein expression changes [130]. Some miRNA profiling studies showed that both sevoflurane and propofol affect miRNA expressions in liver [131], lung [132], and brain [133], all of which has its specific pattern of expression after each anesthetic exposure [131, 132]. Out of 177 expressed miRNAs in mice liver, 46 miRNA expressions were changed after sevoflurane or propofol exposure [131]. Especially, there was significant difference in the expression of miR-142-3p, miR-29a and miR-378 after sevoflurane and propofol exposure [131]. In mice lung, 20 miRNA expressions were significantly altered after sevoflurane exposure when compared to the controls and 16 miRNA expressions were changed after 4% sevoflurane exposure with specific expression patterns [132]. Also, 14 miRNAs were significantly different after sevoflurane and propofol exposure in mice hippocampi [133]. Hence, different anesthetics that induce unique changes in miRNA expression patterns in organs may have specific effects. Therefore, post-transcriptional factors such as miRNAs that may control the regulation of gene expression are expected to play a crucial role in the biological effects of anesthetics.

Previous reports revealed that inhalational and intravenous anesthesia affect disease outcomes via miRNAs. Sevoflurane exerts hepato-protective effects by inducing miR-9-5p expression in ischaemia-reperfusion injury. miR-9-5p targets nuclear factor-kappa B (NF-κB) 3, coding for p65, which is a key protein in the NF-κB signaling pathway. Sevoflurane inhibits the NF-κB signaling pathway and protects the liver from ischaemia-reperfusion injury by increasing miR-9-5p expression [134]. Sevoflurane also ameliorates systemic inflammation in acute lung injury model through miR-155 downregulation [135]. Propofol inhibits lipopolysaccharide-induced neuroinflammation partly by decreasing tumor necrosis factor-α, interleukin-6, and nitric oxide by miR-155 suppression [136]. Propofol may also have a therapeutic effect in suppressing sepsis-induced renal injury by activating miR-290-5p and the subsequent inhibition of C-C motif chemokine ligand 2 and its downstream pathways [137].

### Anesthetics, miRNAs and immune function

Several research revealed that both anesthesia and miRNA varied the immune response in vitro and in vivo. Natural killer (NK) cells are an early cellular defense in the immune system against cancer, which is regulated by miR-181. miR-181 promotes the differentiation of NK cells by targeting Nemo-like kinase and also suppresses the upstream of interferon translation during NK cell activation [138]. Inhalational anesthetics can suppress NK cell activity [139–141], recruitment of macrophages [142] and dendritic cells [143], and cause helper T (Th) polarization from an anti-tumor phenotype (Th1) to a cancer-promoting phenotype (Th2) [144]. In contrast, propofol can increase cytotoxic T lymphocyte (anti-tumor) activity [145], and also exhibits anti-inflammatory and anti-oxidative properties through inhibiting cyclooxygenase-2 and prostaglandin E2 [146]. Multiple receptors on immune cells can be also affected by anesthetics in a wide range of immune function [147]. Previous in vivo studies have shown that inhalational anesthetics reduce NK cytotoxic activity in peripheral blood [139], the number of peripheral leukocytes [142], and alveolar macrophages [148]. However, some clinical studies showed that the choice of sevoflurane or propofol did not show significant difference in circulating percentage of NK cell [149], cytotoxic T lymphocytes [149], regulatory T cells (Treg) [150], and Th1/Th17 ratio in breast cancer surgery [150]. Although miRNAs can cause the differentiation of immune cells and indirectly modulate anti-cancer immunity, how anesthetics affect miRNAs and then indirectly change anti-cancer immune function remain unknown.

### Anesthetics, cancers and miRNAs

Inhalational anesthetics increase the expression of cellular mediators that promote proliferation and migration of cancer cells [151–153]. Sevoflurane promotes the proliferation of glioma stem cells and may increase postsurgical recurrence by upregulation of hypoxia-inducible factor-1α (HIF-1α) and vascular endothelial growth factor [154]. The increased HIF-1α correlates with cancer progression and could serve as a potential therapeutic target in cancer patients [155]. However, sevoflurane may also suppress malignant progression in some cancer cell types through a decreased release of matrix metalloproteinase-2 (MMP-2) and MMP-9, partly due to inactivation of the p38 mitogen-activated protein kinase signaling pathway in lung adenocarcinoma cells, resulting in anti-invasion and anti-migration effects [156]. Propofol was reported to have anti-cancer effects in several tumor cells with increasing apoptosis and reducing cell proliferation [157–159]. Propofol also reduces the level of MMP by inhibiting NF-κB pathways, migration, and invasion in breast cancer [160].

**Fig. 2:**
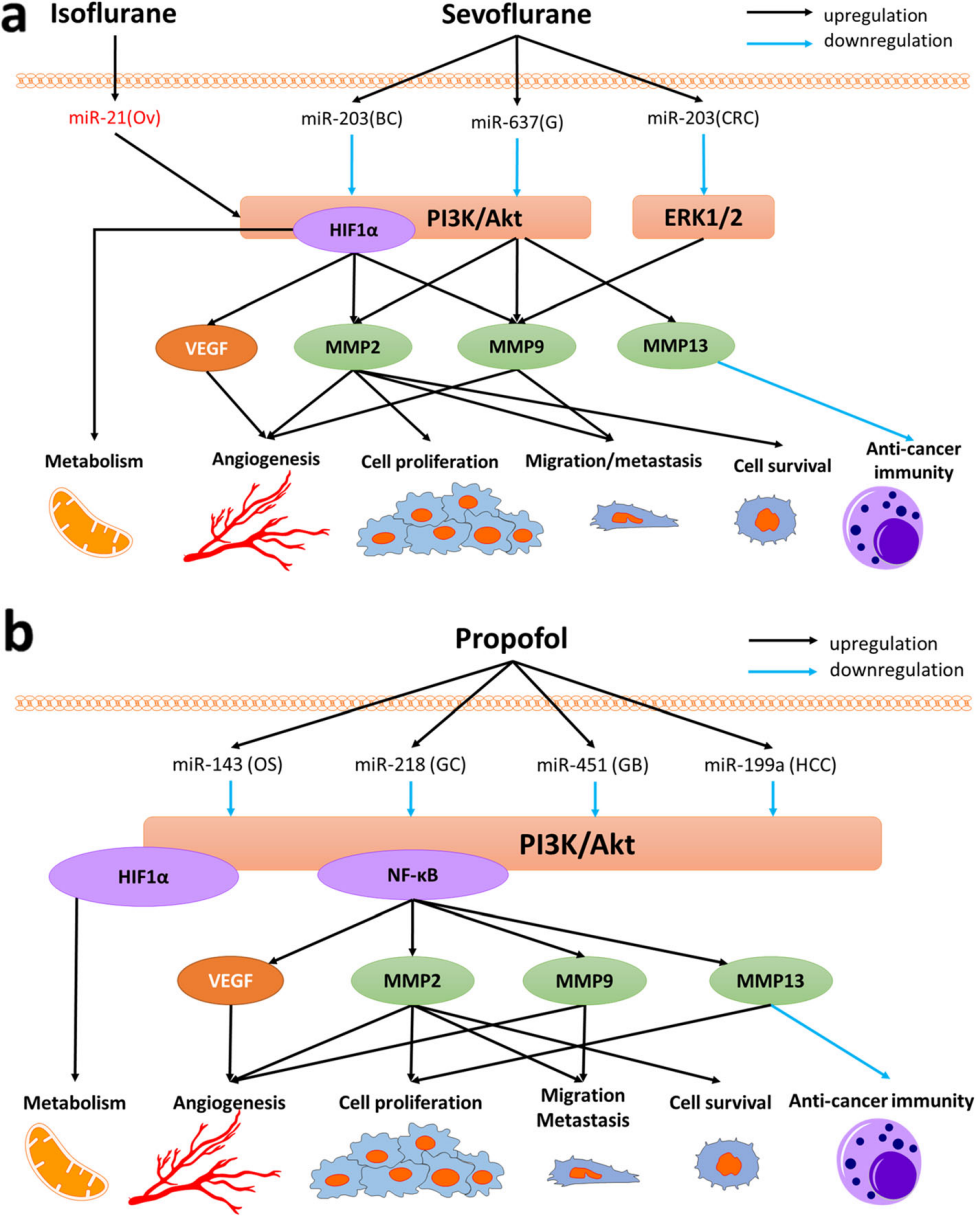
Anesthetic affect pro- and anti-cancer miRNA expressions leading to cancer biology changes. **a**. Inhalational anesthetic reagents modulate miRNA expressions. Isoflurane exposure to ovarian cancer cells increases glycolysis via PI3K/Akt pathway and HIF-1α by miR-21 upregulation, one of oncogenes. Sevoflurane inhibits cancer cell proliferation via PI3K/Akt pathway with miR-203 upregulation in breast cancer cells. In CRC, sevoflurane reduces cancer invasion and migration via the suppression of ERK pathway and MMP-9 by miR-203 upregulation. For glioma cells, sevoflurane increases miR-637 expressions resulting in the decrease of cancer migration and invasion via Akt pathway inhibition. **b**. Propofol exposure alters anti-cancer miRNAs. Propofol upregulates miR-143 in osteosarcoma cells, decreasing cancer cell proliferation by MMP-13 inhibition via PI3K/Akt pathway. In gastric cancer cells, propofol exposure induces apoptosis via PI3K/Akt pathway and MMP-2 by miR-218 upregulation whereas propofol causes apoptosis by miR-451 in glioblastoma cells. For hepatocellular carcinoma, propofol decreases MMP-9 expression by PI3K/Akt pathway suppression via miR-199a upregulation. Ov: ovarian cancer cell, BC: breast cancer cell, G: glioma, CRC: colorectal cancer, OS: osteosarcoma, GC: gastric cancer, GB: glioblastoma, HCC: hepatocellular carcinoma, PI3K/Akt: Phosphatidylinositol 3-kinase/protein kinase B, ERK1/2: extracellular signal-regulated kinases 1 and 2, HIF-1α: hypoxia-inducible factors 1α, NF-κB: nuclear factor-κB, VEGF: vascular endothelial growth factor, MMP: matrix metallopeptidases

Suppressing breast cancer cell proliferation by arresting the cell cycle at the G1 phase was documented via upregulated miR-203 by sevoflurane [161] (Fig. 2a). In colorectal cancer cell, sevoflurane inhibits cancer invasion and migration by downregulation of ERK (extracellular signal-regulated kinases) pathway and MMP-9 via miR-203 upregulation [162]. The inhibitory effects of sevoflurane on glioma cell migration and invasion are mediated by the upregulation of miR-637, which was due to the suppression of Akt (protein kinase B) expression and activity [163]. Isoflurane enhances the cancer malignancy with miR-21 upregulation with the increase of glycolysis product and the related enzymes through Akt phosphorylation [164].

During tumor development, MMPs digest various extracellular matrix components, including proteoglycans, collagen and fibronectin, and provide a favourable environment for primary tumorigenesis. MMPs induce tumor cell migration by removing sites of adhesion, exposing new sites for tumor growth, and releasing pro-cancer factors from the extracellular matrix. Propofol inhibits cell proliferation and MMP-2 expression, and induces apoptosis by miR-218 upregulation in gastric cancer [165] and miR-451 upregulation in glioblastoma cell line [166] (Fig. 2b). Propofol was also reported to decrease hepatocellular carcinoma invasiveness partly due to MMP-9 suppression by miR-199a upregulation [167] and inhibit osteosarcoma cell proliferation through affecting miR-143 expression, which regulates MMP-13 protein expression [159].

### miRNAs as cell-to-cell communication factors induced by anesthetics

**Fig. 3:**
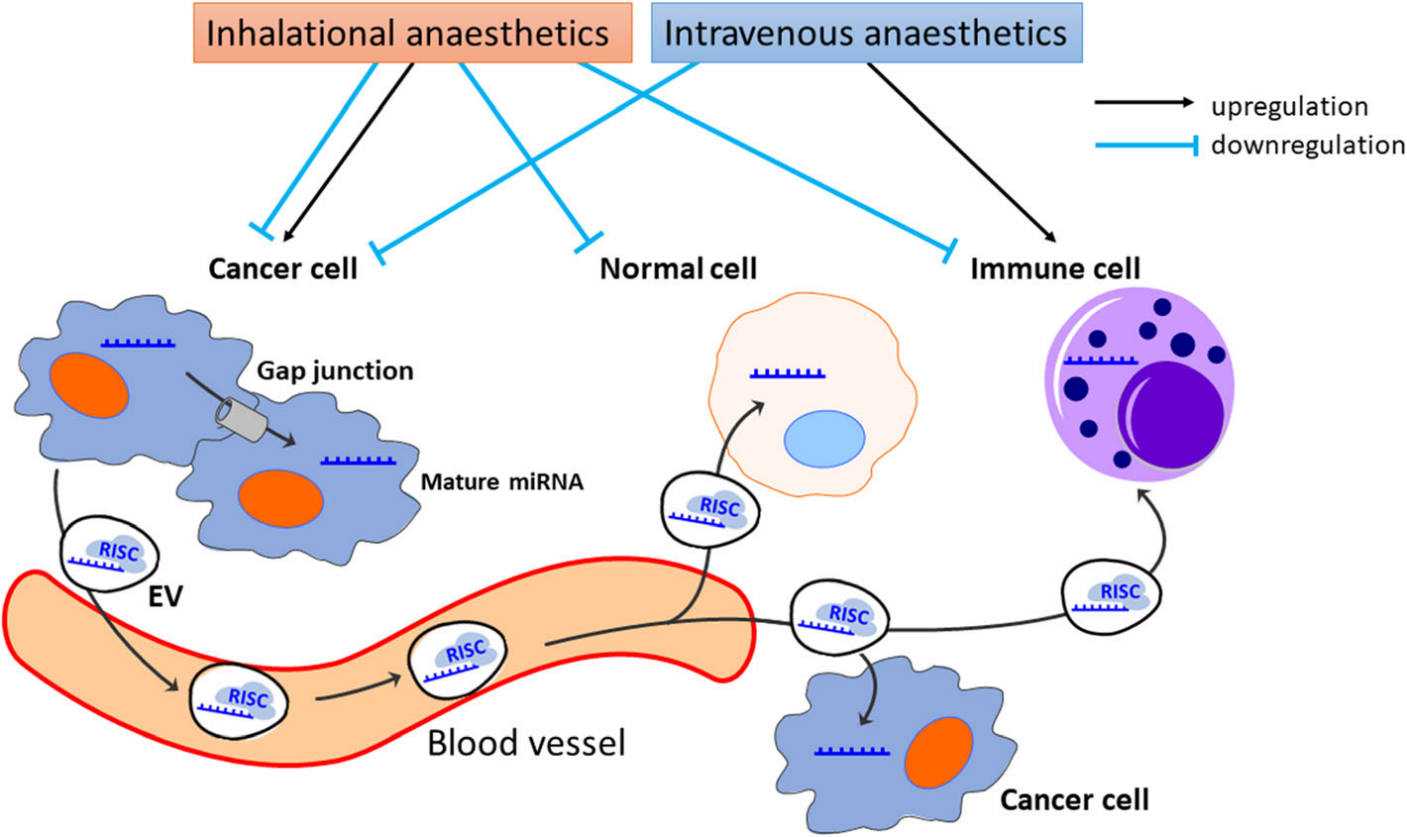
The anesthetic effects on cancer cell biology, anti-cancer immunity, and cell-to-cell communication via miRNAs. Anesthetic exposure during cancer surgery can affect normal cells, anti-cancer immune cells and cancer cells directly or indirectly via miRNA expression changes. Mature miRNA induced by anesthesia inhibits its target protein expression in the affected cell and the adjacent cell with gap junction. Also, miRNA with RISC is released as EV from cells by exocytosis or in microvesicles, which enable miRNA to alter the target protein expressions in the distant cells including normal cells, anti-cancer immune cells and other cancer cells: ‘cell-to-cell communication’. miRNA: micro RNA, RISC: RNA-induced silencing complex, EV: extracellular vesicle

Cell-to-cell communication is critical for regulating biological functions (Fig. 3). The communication occurs directly by cell-to-cell contact, e.g., via cell surface ligand–receptor interactions and gap junctions, and also indirectly through secretion of mediators such as cytokines and hormones [168]. Extracellular vesicles (EVs) are important indirect cell-to-cell communication carriers. Circulating EVs are found in body fluids such as saliva, blood, serum, and urine, and all are in enriched with mRNAs and miRNAs. Propofol may, at least in part, have anti-cancer effects via miRNA-mediated cell-to-cell communication [169]. Propofol-regulated miRNAs inhibit cellular signaling pathways via their downstream effectors that are involved in cell proliferation, migration, and epithelial-mesenchymal transition of tumor cells. In this way, propofol can induce apoptosis of colorectal cancer cells. However, no clinical evidence indicates that miRNAs in circulating EVs can affect cancer recurrence and hence long-term outcomes and further studies are needed.

### Clinical relevance

Both inhalational and intravenous anesthetics are widely used for cancer surgery. Inhalational anesthetics are increasingly associated with worse cancer outcomes compared to intravenous anesthetics used during cancer surgery. Some retrospective clinical data showed that propofol-based total intravenous anesthesia (TIVA) provides better outcomes in breast, colon, rectal, gastric, and oesophageal cancers [170–173]. Conversely, Kim et al. reported that recurrence and overall survival following TIVA for breast cancer surgery are not significantly different compared to surgery under inhalational anesthesia [174]. Inhalational anesthesia might be comparable to TIVA with regard to overall survival in patients with various cancers [175, 176]. A large retrospective study indicated no significant relation to anesthetic type and recurrence free survival (hazard ratio (HR), 0.96; 95% CI, 0.69–1.33, *p* = 0.782) nor overall survival among breast cancer patients with propensity matching (any inhalational anesthetics vs propofol-based TIVA, *n* = 1766 each, HR, 0.96; 95% CI, 0.69–1.33, *p* = 0.805) [176]. A study among high-grade glioma patients showed that sevoflurane did not change progression-free survival, but worsen the mortality (the risk of death after sevoflurane use during surgery, HR, 1.66; 95% CI, 1.08–2.57; *P* = 0.022) and overall survival among patients with reduced performance status (median of overall survival, sevoflurane, *n* = 154 vs propofol, *n* = 140, 15 months vs 11 months; *P* = 0.017). Another study showed that propofol-based TIVA or any inhalational anesthetics had no effects on cancer recurrence nor mortality in non-small lung cell carcinoma patients with the matching performance status (any inhalational anesthetics vs propofol-based TIVA, *n* = 181 each, cancer recurrence, HR 1.310; 95% CI, 0.841–2.041; *p* = 0.233, mortality, HR 0.902; 95% CI, 0.643–1.265; *p* = 0.551) [175]. Furthermore, the consensus statement derived from the BJA Workshop on Cancer and Anesthesia stated that there is insufficient evidence to support any change in current clinical practice [177], and which anesthetic techniques are suitable for cancer surgeries remains unclear. In addition, at this moment, the clinical impact of miRNA alterations on cancer outcomes is still unclear. Therefore, the anesthetic mechanisms on cancer cell biology are still subjected to investigate further.

## Conclusion

Inhalational and intravenous anesthetics have both pro- and anti-cancer effects through various pathways by adjustment of miRNAs. Their effects vary depending on the cancer cell type. Although our understanding of the potential influence of anesthetics on cancer cell biology has been greatly increased by laboratory investigations, a limited number of publications of the effects of anesthetics on cancer cells by miRNA expression changes have been published but the impact of those data on clinical outcomes remains largely unknown. Further studies are needed to integrate basic scientific findings and clinical data related to the effects of anesthetics on cancer cell progression, anti-cancer immunity, cell-to-cell communication, and clinical outcomes via miRNA modulation. Prospective clinical trials are ongoing to investigate the effects of anesthetics on cancer recurrence and survival. Undoubtedly, bridging the gap between basic research findings and clinical data towards evidence-based treatment reminds a challenge, but even a small progress of that will have enormous potential in improving patient outcomes. To this end, the molecular effects including miRNAs of anesthetics on cancer cell “behavioral” changes are needed to investigate further and ultimately optimal anesthetic regimens can be implemented for cancer surgery.

## Data Availability

The datasets used and/or analysed during the current study available from the corresponding author on reasonable request.

## Abbreviations

miRNAs: microRNA
XIAP: X-linked inhibitor of apoptosis
PD-L1: Programmed death-ligand 1
BCL2: B-cell lymphoma 2
DNAJB1: DnaJ homolog subfamily B member 1
CTNNB1: Catenin Beta 1
FOXP1: Forkhead box protein P1
HDAC1: Histone deacetylase 1
eNOS: Endothelial nitric oxide synthase
NSAIDs: Non-steroidal anti-inflammatory drugs
ARDS/ALI: Acute respiratory distress syndrome/acute lung injury
POCD: Postoperative cognitive dysfunction
DVT: Deep venous thrombosis
AKI: Acute kidney injury
pro-BNP: Pro-brain natriuretic peptide
CXCL: Chemokine (C-X-C motif) ligand
NK: Natural killer
NF-κB: Nuclear factor-kappa B
HIF-1α: Hypoxia-inducible factor-1
MMP: Matrix metalloproteinase
ERK: Extracellular signal-regulated kinases
Akt: Protein kinase B
EV: Extracellular vesicle
TIVA: Total intravenous anesthesia
